# Suicide Deaths During the COVID-19 Stay-at-Home Advisory in Massachusetts, March to May 2020

**DOI:** 10.1001/jamanetworkopen.2020.34273

**Published:** 2021-01-21

**Authors:** Jeremy Samuel Faust, Sejal B. Shah, Chengan Du, Shu-Xia Li, Zhenqiu Lin, Harlan M. Krumholz

**Affiliations:** 1 Division of Health Policy and Public Health, Department of Emergency Medicine, Brigham and Women’s Hospital, Harvard Medical School, Boston, Massachusetts; 2 Department of Psychiatry, Brigham and Women’s Hospital, Harvard Medical School, Boston, Massachusetts; 3 Center for Outcomes Research and Evaluation, Yale New Haven Hospital, Yale University School of Medicine, New Haven, Connecticut; 4 Center for Outcomes Research and Evaluation, Yale New Haven Hospital, New Haven, Connecticut

## Abstract

This cohort study evaluates the association between the coronavirus disease 2019 (COVID-19) stay-at-home orders and suicide deaths in Massachusetts.

## Introduction

Many policy makers believe that shelter-in-place or stay-at-home policies could cause an increase in what are known as deaths of despair. While increases in psychiatric stressors during the coronavirus disease 2019 (COVID-19) pandemic have been reported, it is presently unknown whether suicide rates similarly changed during stay-at-home periods.^1,2^

## Methods

In this cohort study, we assembled suicide death data for persons aged 10 years and older from the Massachusetts Department of Health Registry of Vital Records and Statistics from January 2015 through May 2020. This study was not subject to institutional review board approval or the requirement for informed consent because it used public data. The Strengthening the Reporting of Observational Studies in Epidemiology (STROBE) reporting guideline was followed.

We used autoregressive integrated moving average (ARIMA) and seasonal ARIMA models to analyze suicide deaths in Massachusetts, with yearly population as the covariate. We used the Akaike information criterion (AIC) to select the best model. We plotted suicide deaths during each month of 2020 for which adequate data exist (January to May). To be conservative, we plotted an alternative scenario in which the number of deaths pending investigation by the state medical examiner exceeding the corresponding monthly averages from 2015 to 2019 were ascribed to suicide. Incident rates and rate ratios for the stay-at-home period (ie, March to May) and the corresponding period in 2019 were determined. Statistical significance was set at *P* < .05, and all tests were 2-tailed. Analysis was conducted with R version 4.0.2 (R Project for Statistical Computing) and SAS version 9.4 (SAS Institute).

## Results

During the pandemic period, the incident rate for suicide deaths in Massachusetts was 0.67 (95% CI, 0.56-0.79) per 100 000 person-months vs 0.80 (95% CI, 0.68-0.93) per 100 000 person-months during the corresponding period in 2019 (incident rate ratio, 0.84; 95% CI, 0.64-1.00). Because data for 2019 and 2020 are preliminary, a sensitivity analysis including all deaths still pending final cause adjudication as of November 14, 2020, was performed. The addition of the 47 deaths pending cause determination occurring from March through May 2020 and the 32 cases still pending determination from the corresponding period in 2019 did not change these findings. The conservative assumption that all pending investigations for March to May were suicides yielded an incident rate of 0.89 (95% CI, 0.77-1.03) deaths per 100 000 person-months for 2020 and 0.95 (95% CI, 0.83-1.10) deaths per 100 000 person-months for 2019 (incident rate ratio, 0.94; 95% CI, 0.76-1.15).

The number of suicide deaths during the stay-at-home period did not deviate from projected expectations using either preliminary data or an alternate scenario in which deaths pending investigation that exceeded the average remaining number of deaths that occurred during the corresponding period in 2015 to 2019 were ascribed to suicide (Figure). Decedent age and sex demographic characteristics were unchanged during the pandemic period compared with those during 2015 to 2019 (Table).

**Figure.**
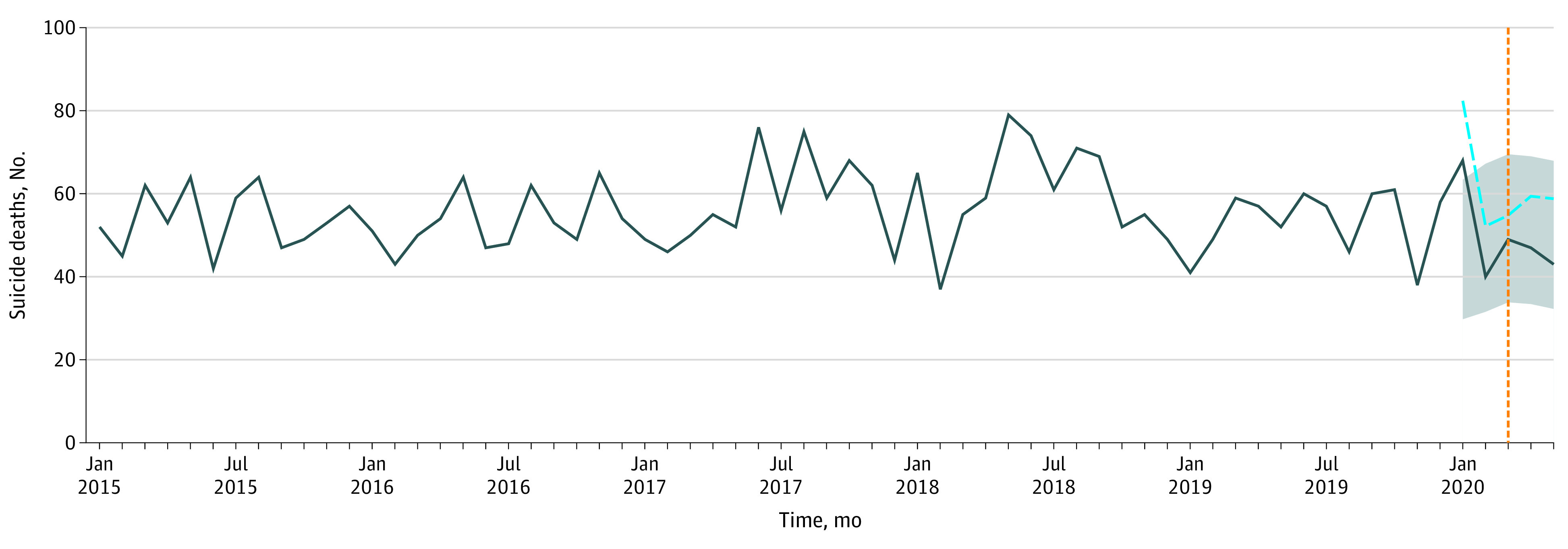
Suicide Deaths in Massachusetts From January 2015 Through May 2020 The solid line indicates raw suicide death counts; the dashed blue line, raw suicide deaths plus deaths pending investigation by the state medical examiner that are in excess of monthly averages of active pending investigations during the corresponding months from 2015 to 2019; gray shaded area, projected range of suicide deaths expected to occur during 2020 using the seasonal adjusted model; vertical orange line, the start of the stay-at-home period.

**Table.** Age and Sex Demographic Characteristics Among Individuals Who Died by Suicide in Massachusetts

Year	Deaths, No./total No. (%)		Mean age, y		
	Women	Men	Women	Men	All
2015	44/180 (24.4)	136/180 (75.6)	47.6	47.5	47.6
2016	39/169 (23.1)	130/169 (76.9)	47.5	44.7	45.3
2017	38/157 (24.2)	119/157 (75.8)	49.7	44.2	45.5
2018	45/193 (23.3)	148/193 (76.7)	46.3	46.3	46.3
2019	37/166 (22.3)	129/166 (77.7)	44.4	50.0	48.8
2020	34/139 (24.5)	105/139 (75.5)	44.7	49.9	48.6

## Discussion

The stable rates of suicide deaths during the stay-at-home advisory in Massachusetts paralleled findings following ecological disasters.^3^ Early in the outbreak, social distancing–created stressors may have been offset by a sense of shared purpose in flattening the curve. There were efforts at bolstering connections through video platforms; anticipation regarding governmental support, including unemployment benefits and stimulus aid; and mental health awareness campaigns about the risks of isolation, loneliness, and despair that could accompany the public health imperative of physical distancing. One limitation to this study is its reliance on cause-of-death adjudication. However, unlike other causes of death, every suicide is investigated by a medical examiner, rendering resulting death certificates more reliable than many other causes. As the pandemic persists, uncertainty about its scope and economic impact may increase.^4^ Suicide risk often increases with rising unemployment and related strains, access to firearms, substance use, and interpersonal violence.^5^ Individuals with serious mental illness whose illness worsens may also contribute to related morbidity and mortality. However, our data suggest that an increase in suicide deaths in Massachusetts was not associated with the stay-at-home advisory. Moving forward, effective prevention efforts will require comprehensive attention to the full spectrum of mental health services.
